# Editorial: The Chemistry of Imaging Probes

**DOI:** 10.3389/fchem.2018.00253

**Published:** 2018-06-26

**Authors:** Lorenzo Tei, Zsolt Baranyai

**Affiliations:** ^1^Dipartimento di Scienze e Innovazione Tecnologica Università del Piemonte Orientale “Amedeo Avogadro”, Alessandria, Italy; ^2^Bracco Imaging (Italy) Colleretto Giacosa, Italy

**Keywords:** molecular imaging probes, chelating ligands, contrast agents, coordination chemistry, thermodynamic and kinetic stability

Over the past decades, the field of molecular imaging (MI) has been rapidly growing involving multiple disciplines such as medicine, biology, chemistry, pharmacology and biomedical engineering. An *in vivo* MI protocol non-invasively provides visual and quantitative information on normal or pathological processes at the cellular or sub-cellular level. Currently, molecular imaging can be performed in the clinical environment via Magnetic Resonance Imaging (MRI), Optical Imaging (OI), Positron Emission Tomography (PET), Single-Photon Emission Computed Tomography (SPECT), Computed Tomography (CT) and Ultrasound (US). Each of these imaging modalities has its own advantages and disadvantages, and therefore, a multimodal approach combining two techniques is often adopted to generate complementary anatomical and functional information of the disease (Anderson and Lewis, 2017).

The key requirement for any molecular imaging procedure is a chemical agent, called the imaging probe. This probe typically consists of two chemical entities: one that produces a signal for imaging and another, a targeting vector, which enables the probe to specifically visualize, characterize and quantify biological processes in living systems. The optimization of the probes involves several different aspects ranging from the design of imaging units that exhibit enhanced sensitivity and lower toxicity, to the control of the structural and electronic features of the targeting moiety responsible for the molecular recognition (Long and Wong, 2015). In this research topic we present a series of original research and review articles that summarize the chemistry of molecular imaging, both in terms of optimization of the MI probe and in terms of application of different imaging techniques such as MRI, PET/SPECT, OI, and US.

The optimization of MRI contrast agents (CAs) is pursued to obtain more stable, safer and more efficient probes for potential application in molecular imaging. In particular, two dinuclear Gd(III) complexes and their relaxometric characterization have been reported by Leone et al. demonstrating that a significant contribution of second sphere water molecules is responsible for the strong relaxivity enhancement observed over a large range of magnetic field strengths. Regarding Mn(II) complexes of 12-membered macrocyclic ligands as a promising alternative to Gd-based CAs, Tircsó et al. have reviewed the relationship between ligand structure and physicochemical properties of the complexes. The rigidity of the macrocycle and proper selection of the donor atoms in the sidearm are the key structural features that control the thermodynamic and the dissociation kinetic properties of these macrocyclic Mn(II) complexes.

The synthesis, characterization and handling of Eu(II)-containing complexes have been reviewed by Basal and Allen showing how these air-sensitive probes can provide an intriguing and promising approach to new MRI molecular imaging procedures. The review especially focuses on the reduction of Eu(III) to produce Eu(II)-containing complexes and on the handling of Eu(II)-containing samples to prevent oxidation and obtain accurate data.

Chemical Exchange Saturation Transfer (CEST) continues to attract increasing attention as an alternative MR imaging modality to *T*_1_ shortening agents. A contribution from Farashishiko et al. reports on an attempt to improve the sensitivity of these agents by incorporating a large payload of paraCEST agents into a reverse-assembled nanocapsule. Unfortunately, the result was not an amplification of the CEST effect, but quenching of the signal, attributed to an increase of the transverse relaxation rate of chelate protons caused by slow molecular tumbling of the complex in the nanosized system.

^19^F-containing imaging probes can be advantageously employed in MRI and Magnetic Resonance Spectroscopy (MRS) because virtually there is no background ^19^F MR signal *in vivo*. In the present topic, theories and strategies of improving the sensitivity of ^19^F probes with paramagnetic metal ions have been reviewed by Peterson et al. In particular, the paper focuses on a theory that predicts the impact of certain molecular parameters on the sensitivity of fluorine-based probes.

The *in vivo* application of MRI—theranostic probes whose activation is induced by ultrasound, thermal or mechanical effects (sonosensitive probes) has been reviewed by Garello and Terreno. This work shows how these probes can be used for real-time monitoring of triggered drug release. These liposome based nano/microvesicles are composed of a biocompatible membrane responsive to US and an aqueous core loaded with a MRI probe and a therapeutic agent. A significantly better therapeutic effect was observed using US triggered drug release in comparison to traditional therapies.

The investigation of the luminescence properties of a Tb(III)-DOTA-calix[4]arene derivative was carried out by Mayer et al. with the aim to design dual MR/optical imaging probes. The paper shows how the calix[4]arene core with its four aromatic rings acts as an effective sensitizer of Tb-centred luminescence but different substituents on the lower rim can cause micellar aggregation leading to a significant decrease in the intensity of Tb(III) luminescence.

A dual-modality iron oxide nanoplatform for *in vivo* targeted SPECT and MRI investigation of tumor vascularization has been developed by Tsoukalas et al. The NPs were coated with ^99m^Tc radiolabeled Bevacizumab (BCZM) monoclonal antibodies (specific affinity to vascular endothelial growth factor—VEGF-A—receptor). Initial *in vivo* SPECT and MRI studies revealed the suitability of Fe_3_O_4_-DMSA-SMCC-BCZM for targeted dual-modality imaging.

The use of conjugates comprised of more than one targeting biomolecule (multimers) is a well-established and straightforward way to enhance the target affinity and uptake of radiotherapeutics. For this purpose, Wurzer et al. designed a DOTA-tetraphosphinate chelator (DOTPI) possessing four terminal carboxylic acid moieties to obtain tetrameric bioconjugates. The favorable properties of the ^177^Lu labeled tetrameric DOTPI(PSMA)_4_ conjugates for targeted radionuclide therapy have been demonstrated in *in vitro* and *in vivo* studies.

In order to rationalize the influence of metal ion contamination [e.g., Ti(IV), Fe(III), Cu(II), Zn(II) or Al(III)] on ^68^Ga labeling, the physicochemical properties of Ga(TRAP) and Fe(TRAP) (TRAP = triazacyclononane phosphinic acid) were investigated in detail by Vágner et al. Equilibrium and kinetic data showed that the stability constants and the dissociation rates of Ga(III)- and Fe(III)-complexes are very similar. However, the slower formation of Fe(TRAP) allows selective labeling of TRAP with ^68^Ga(III) even in presence of Fe(III) contamination in the eluate.

## Author contributions

The authors have made a substantial, direct, and intellectual contribution to the work, and approved it for publication.

### Conflict of interest statement

The authors declare that the research was conducted in the absence of any commercial or financial relationships that could be construed as a potential conflict of interest.
